# User Experience of Interactive Technologies for People With Dementia: Comparative Observational Study

**DOI:** 10.2196/17565

**Published:** 2020-08-05

**Authors:** Luis Duarte Andrade Ferreira, Henrique Ferreira, Sofia Cavaco, Mónica Cameirão, Sergi Bermúdez i Badia

**Affiliations:** 1 NOVA LINCS, Dep de Informática Faculdade de Ciências e Tecnologia Universidade Nova de Lisboa Lisboa Portugal; 2 Madeira Interactive Technologies Institute Universidade Madeira Funchal Portugal; 3 Faculdade de Ciências Exatas e da Engenharia Universidade da Madeira Funchal Portugal

**Keywords:** dementia, technology, interaction, psychomotor performance, equipment safety, costs and cost analysis, user-computer interface

## Abstract

**Background:**

Serious games (SGs) are used as complementary approaches to stimulate patients with dementia. However, many of the SGs use *out-of-the-shelf* technologies that may not always be suitable for such populations, as they can lead to negative behaviors, such as anxiety, fatigue, and even cybersickness.

**Objective:**

This study aims to evaluate how patients with dementia interact and accept 5 *out-of-the-shelf* technologies while completing 10 virtual reality tasks.

**Methods:**

A total of 12 participants diagnosed with dementia (mean age 75.08 [SD 8.07] years, mean Mini-Mental State Examination score 17.33 [SD 5.79], and mean schooling 5.55 [SD 3.30]) at a health care center in Portugal were invited to participate in this study. A within-subject experimental design was used to allow all participants to interact with all technologies, such as HTC VIVE, head-mounted display (HMD), tablet, mouse, augmented reality (AR), leap motion (LM), and a combination of HMD with LM. Participants’ performance was quantified through behavioral and verbal responses, which were captured through video recordings and written notes.

**Results:**

The findings of this study revealed that the user experience using technology was dependent on the patient profile; the patients had a better user experience when they use technologies with direct interaction configuration as opposed to indirect interaction configuration in terms of assistance required (*P*=.01) and comprehension (*P*=.01); the participants did not trigger any emotional responses when using any of the technologies; the participants’ performance was task-dependent; the most cost-effective technology was the mouse, whereas the least cost-effective was AR; and all the technologies, except for one (HMD with LM), were not exposed to external hazards.

**Conclusions:**

Most participants were able to perform tasks using *out-of-the-shelf* technologies. However, there is no perfect technology, as they are not explicitly designed to address the needs and skills of people with dementia. Here, we propose a set of guidelines that aim to help health professionals and engineers maximize user experience when using such technologies for the population with dementia.

## Introduction

### Background

The difference between serious games (SGs) or transformational games [1] and entertainment video games is that SGs are software apps with a defined goal that goes beyond pure entertainment [2]. In the field of health care, SGs have been developed and used for a variety of purposes, such as training and simulation [3], diagnosis and therapy [4-6], education [7], and other purposes [8]. In recent years, there has been a growing interest in using games to target health conditions, such as stroke [9], Parkinson disease [10], and autism [11]. To a lesser extent, SGs have also been used with people with dementia [12].

Dementia is a neurocognitive disorder [13,14], which impairs cognitive and emotional behaviors, such as memory, language, problem-solving, anxiety, irritability, visuospatial issues, gait and balance-related issues, and other dementia-related aspects [13-18]. Although Alzheimer disease is the most common form of dementia [15], there are other types of dementia, such as vascular dementia [19], Lewy body dementia [20], frontotemporal dementia [21], and mixed dementia [22]. In general, the disease can profoundly affect the carriers, family members, professional caregivers [23], and health care systems [24]. It is estimated that over 35 million individuals worldwide have dementia and that dementia-related expenses reached approximately US $818 billion in 2015 [25]. Portugal alone had expenses ranging between US $1652.8 million and US $2120.4 million in 2009 [24].

Although there are pharmaceutical approaches to treat dementia, these often have side effects, or their desired outcome is only temporary [26]. In addition, the development of new drugs is not only expensive but also time-consuming, as it needs to go through several scientific trials before being approved for human use [27]. As a result, the search for alternative methods, such as SGs, has greatly increased [28].

### SGs and Technologies

SGs for people with dementia have been developed as an assistive tool to promote physical, cognitive, and emotional stimulation, leading to a better quality of life [12,29]. Moreover, a novel SG can be used to assess cognitive decline at the early stages (or elevated risk) of dementia [30-32]. Investment in such computer apps can provide an opportunity to reduce institutional health care costs and enhance the quality of life of both family caregivers and people with dementia [33].

Many platform strategies have been considered to develop dementia-related SG apps. Some technologies rely on indirect interactions, such as (PCs) [34], or conventional entertainment systems, such as the Nintendo Wii system [35]. Other technologies are based on direct interaction, such as augmented reality (AR) [36], touchscreen technology [37-39], and gesture recognition systems, such as leap motion (LM), Kinect, and Bracelet Myo [40].

Indirect interaction technologies require an intermediate device to translate human action into interaction with the virtual environment. Indirect interaction devices use more cognitive resources, as they involve conscious spatial and mental translations to convert real-world movements into virtual actions [41].

Using direct interaction technologies, participants do not have an intermediary device to interact with the virtual environment; participants interact directly with the machines using their bodies [41]. In addition, direct interaction devices require less cognitive resources, as there is no movement translation between the real and virtual worlds as opposed to indirect interaction devices [41].

### Proposed Frameworks to Develop SGs

In terms of the development of SGs, previous studies revealed interesting insights regarding the development process of SG. For example, Brian Winn proposed the *design, player, experience* framework, which depicts the relationship between the designer and the player’s experience [42]. The framework is quite straightforward: the designer designs the game, which is played by the player according to the player’s experience. According to this framework, *play* is mediated by experience. Thus, the player’s experience (social, cultural, cognitive, and experimental background) influences the design of the game.

The study also considers the learning process in using technological devices to play games, as it can also influence users’ game experience. For example, in a recent study, Vallejo et al [43] evaluated the performance of elderly individuals on a set of technologies while performing 2 different tasks. The study concluded that interaction with technology is dependent on the task, the user’s experience, and motivation. In addition, the interaction with technology also depends on how intuitive both hardware and software interfaces are for people with dementia [28]; many high-tech technologies can overwhelm people with dementia on a cognitive level, which can affect the learning curve of handling technology [28,44].

Thus, to enhance the user experience for people with dementia while using novel technology, additional guidelines have been suggested to help developers in designing technologies while addressing the need for people with dementia, such as the responding, enabling, augmenting, failure-free (REAFF) framework [45,46]. The REAFF framework focuses on 4 principles: (1) *responding* (technologies should respond to the needs of people with dementia), (2) *enabling* (technologies should improve the quality of life of people with dementia), (3) *augmenting* (technologies should be able to adapt to the reserved skills of people with dementia), and (4) *failure free* (technologies should be as easy to use as possible without discouraging people with dementia).

Another framework—the virtual reality (VR)-Check framework [47]—has been proposed by Krohn et al [47], who evaluated clinical neuropsychology VR apps for cognitive domain specificity ( specifically, the cognitive domain being targeted by the VR app), ecological relevance (if the VR app focuses on activities of daily living), technical feasibility (if the VR app is compatible with the desired technologies), user feasibility (if the VR app is feasible to the target population), user motivation (if the VR app engages the users), task adaptability (if the VR app can be adjusted, for instance, in terms of difficulty), performance quantification (if the VR app can objectively quantify the participant’s performance), immersive capacities (how immersive is the VR app for participants), training feasibility (if the VR app is suitable to foster cognitive training), and predictable pitfalls (estimating resource-related costs when using the VR app).

### Main Purpose of This Study

Despite the existence of several efforts aimed at providing recommendations to develop SGs [48-50], there is still a lack of usability studies that aim to understand how people with dementia interact and accept different types of technologies to perform specific tasks [51,52]. Although elderly individuals are capable of learning and handling new technologies [53], using novel technology can lead to anxious behaviors among elderly populations [54] or lead to undesirable side effects, such as cybersickness [55] and fatigue [28].

To avoid such behaviors, during the prototype playtest phase, it is essential to record the feedback of each player while interacting with the game, as the experience of one player may differ significantly from the experience of another player [42]. In a recent study, Hackner et al [56] analyzed how people with dementia perform different interaction techniques in a tablet, such as a single tap, swipe, and drag-and-drop gestures. The study identified several interaction issues when performing such interaction techniques and presented different solutions to avoid future problems.

Considering the reported potential of SGs as a complementary approach to stimulate people with dementia, the main goal of this study was to better understand how people with dementia accept and interact with *out-of-the-shelf* technologies and how it influences users’ game experience while performing different activities. Moreover, this study aimed to find the most suitable technology to design a customizable interactive system that can exploit reminiscence and music therapy in people with dementia. We recruited 12 participants with dementia to perform several activities with different technologies to evaluate their performance while answering 6 research questions (RQs):

- RQ1. Is there a relationship between the patient’s profile and user experience?
- RQ2. Is there a relationship between user experience and direct and indirect interaction?
- RQ3. Does any technology elicit more positive or negative emotional responses?
- RQ4. Overall, which technology is better suited for each task?
- RQ5. Which technology is the most cost-effective?
- RQ6. Which technology is less exposed to external hazards?

Following the results of our experiment, we (1) propose a set of guidelines that can help engineers and developers craft better-suited technologies for this population and (2) suggest additional setups of the technologies used to improve user experience in people with dementia.

## Methods

### Participants

We recruited 12 participants, 3 males and 9 females, with mean age, 75.08 (SD 8.07) years; mean Mini-Mental State Examination score, 17.33 (SD 5.79); and mean schooling, 5.55 (SD 3.30). This was a convenience sample, and the recruitment of the participants was performed by psychologists at the Madeiran delegation of the Portuguese Alzheimer’s Association (Table 1). Participants were eligible if they (1) could use upper limbs independently, (2) had an intact hearing, and (3) were in the initial or intermediate stages of dementia. For the last inclusion criteria, we relied on the clinical information available and did not perform any further assessments. The study was approved by the board of the association and followed the standard procedures for research with human participants. Before beginning the experimental trial, all participants (or legal guardians) signed an informed consent form, and permission was granted to film the sessions. After signing the consent form, participants were briefed about (1) the activity objectives and (2) how to handle the technologies. In addition, participants were informed that they could drop out of the experimental trial at any time.

We defined patients’ profiles based on their Mini-Mental State Examination (MMSE) [57] scores, age, and years of schooling. The MMSE scores were assessed before the participation of the experimental trial. Only 5 of the participants reported previous experience with technology. For example, participant 1 had experience using a tablet, whereas participants 11 and 12 had experience with PC. Participants 5 and 7 had experience in handling both PC and tablet.

**Table 1:** Participants’ demographics.

Participants	Genders	Age (years)	MMSE^a^ score	Schooling (year)	Diagnostics
1	Female	70	25	Fourth	Alzheimer disease
2	Female	85	19	Fourth	Alzheimer disease
3	Female	78	18	Third	Vascular dementia
4	Male	81	17	—^b^	Alzheimer disease
5	Male	67	24	Fifth	Frontotemporal dementia
6	Female	74	12	Third	Alzheimer disease
7	Female	71	14	Fourth	Alzheimer disease
8	Male	82	21	Fourth	Lewy body dementia
9	Female	65	11	Sixth	Alzheimer disease
10	Female	88	10	Twelfth	Alzheimer disease and Parkinson disease
11	Female	77	26	Fourth	Alzheimer disease
12	Female	63	11	Twelfth	Frontotemporal dementia

^a^MMSE: Mini-Mental State Examination.

^b^Participant 4 does not have any formal schooling.

### Technologies Used During the Experiment

For each of the following technologies, we selected generic tasks that required different types of interaction, such as (1) manipulating virtual objects, (2) playing musical instruments, (3) moving virtual objects from A to B, and (4) observation of virtual environments.

To run the tasks and technologies, we used a Toshiba Satellite L850-1HZ with Windows 10 64 bit equipped with an AMD Radeon HD 7670 and an Intel Core i7-3630QM with 4 GB RAM. Considering that some technologies require a considerable amount of processing power, a desktop computer running Windows 10 64 bits equipped with a Radeon RX 580 Series graphic card and an Intel Core 17-6700 CPU with 16 GB RAM were used. Five different interaction technologies were used in different combinations and tasks.

#### Indirect Interaction Configurations

**Figure 1:**
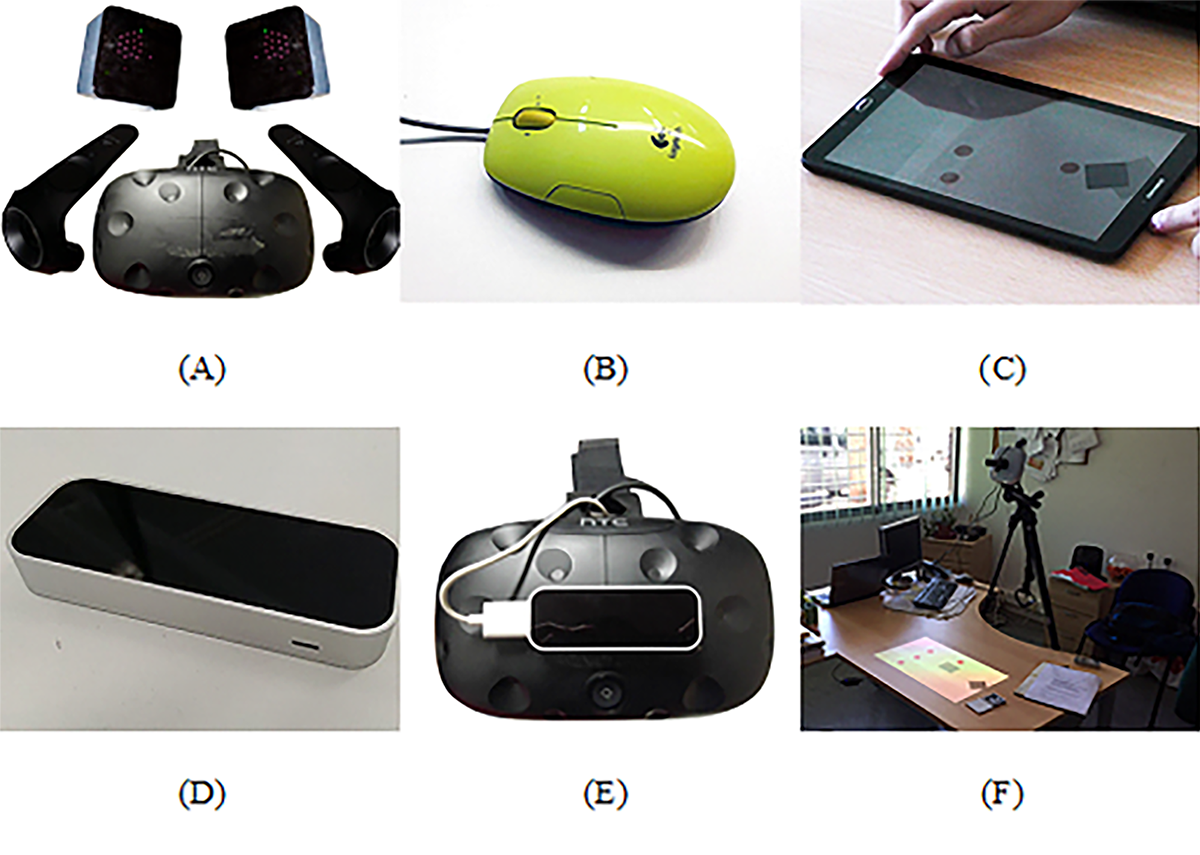
Technologies used during the experimental trial. (A) HMD with Controllers. (B) Mouse. (C) Tablet. (D) Leap Motion. (E) HMD with Leap Motion. (F) Augmented Reality.

Here we present 2 indirect interaction configurations: HMD with controllers and mouse. Each configuration is described below:

1. HTC VIVE with controllers (HMD with controllers): The HTC VIVE technology (HTC) is a set of different technologies that includes a head-mounted display (HMD) and 2 handheld controllers, which are equipped with a trackpad, menu button, system button, trigger button, and grip button. Two base stations were used to track the position and movements of the participant’s head and hands (Figure 1).
2. Mouse: We used a standard USB-powered laser mouse (Logitech LS1 Laser Mouse, Logitech International). The mouse is designed with 3 buttons: left, right, and a wheel button (Figure 1).

#### Direct Interaction Configurations

Here we present 5 direct interaction configurations: HTC-VIVE, tablet, LM, HTC with LM and AR. Each configuration is described below:

1. HTC VIVE (HMD): The HTC VIVE allows the use of the HMD without the controllers to interact with the virtual environments (Figure 1).
2. Tablet: We used a Samsung 9“ Android Tablet (GALAXY, Samsung) that allows interaction inputs, such as tapping and dragging (Figure 1).
3. LM: LM (Motion Control) is an infrared camera–based tracking technology that allows interaction with the virtual environment using hands, fingers, and tools [58] (Figure 1).
4. HTC VIVE with LM (HTC with LM): We added the LM to the HTC VIVE HMD. Thus, participants could interact with the virtual environment not only using head movements but also with their hands (Figure 1).
5. AR: For AR, we developed a projection-based setup that required a projector (LG Inc) and a PlayStation Eye camera (Sony Computer Entertainment Inc), which were attached to a tripod. A physical object with a marker attached to it was used by the participants to interact with the virtual environment. For marker recognition, the Analysis and Tracking System [59] software was used, which allowed the tracking of the physical object (Figure 1).

### Manipulating Virtual Objects

#### LM

**Figure 2:**
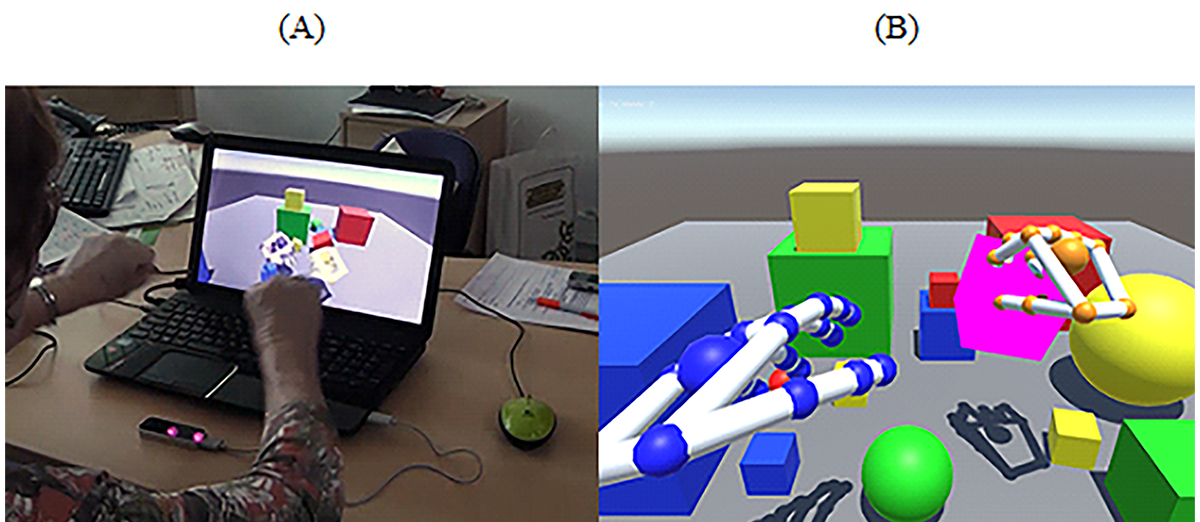
Virtual Playground. (A) Manipulating virtual objects tasks with hands through leap motion. (B) Print screen of the game. Participants had to interact freely with virtual objects.

The playground was developed using the Unity 3D game engine (Unity Technologies) and consisted of a variety of geometrical figures (Figure 2). In this task, participants were required to use hand gestures, such as grabbing, throwing, and lifting, to interact with the geometrical figures. As participants could interact and throw geometrical figures out of their field-of-view, the virtual playground could be reset by tapping the computer’s space bar. The task did not have any music playing in the background, and it did not provide any additional feedback to the participant.

#### HMD With Controllers

**Figure 3:**
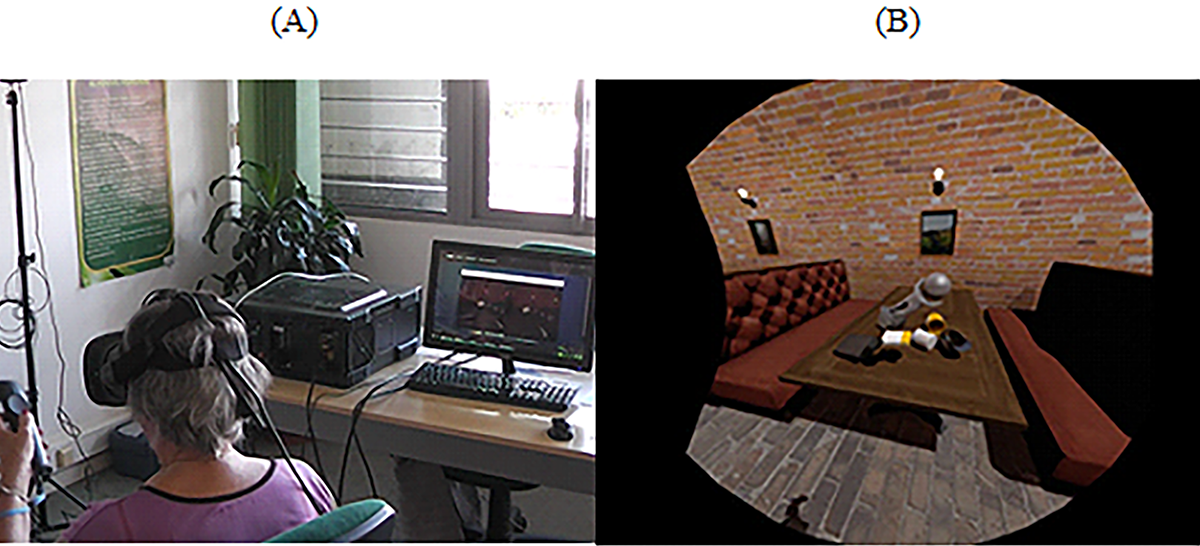
Steam virtual reality home – bar table. (A) Manipulating daily objects using head-mounted display with controllers. (B) Print screen of the task. Participants had to look at and manipulate daily objects that are on the table.

The goal of the task was to manipulate objects that were placed on a table in a virtual music bar with the HMD with controllers (Figure 3). We used a game called *Jam Session* [60], which can be accessed for free on STEAM (Valve Corporation). Several objects such as cups, a doll, a telephone, a clock, a globe, and a book were used (Figure 3). All objects were placed randomly on the table. To perform the task, participants had (1) to use the controllers with both hands; (2) grab objects by pressing the trigger button on the back of the controller; and (3) rotate, throw, or place the object wherever they wanted. The task did not have any music playing in the background, and it did not provide any additional feedback to the participant.

#### HMD With LM

**Figure 4:**
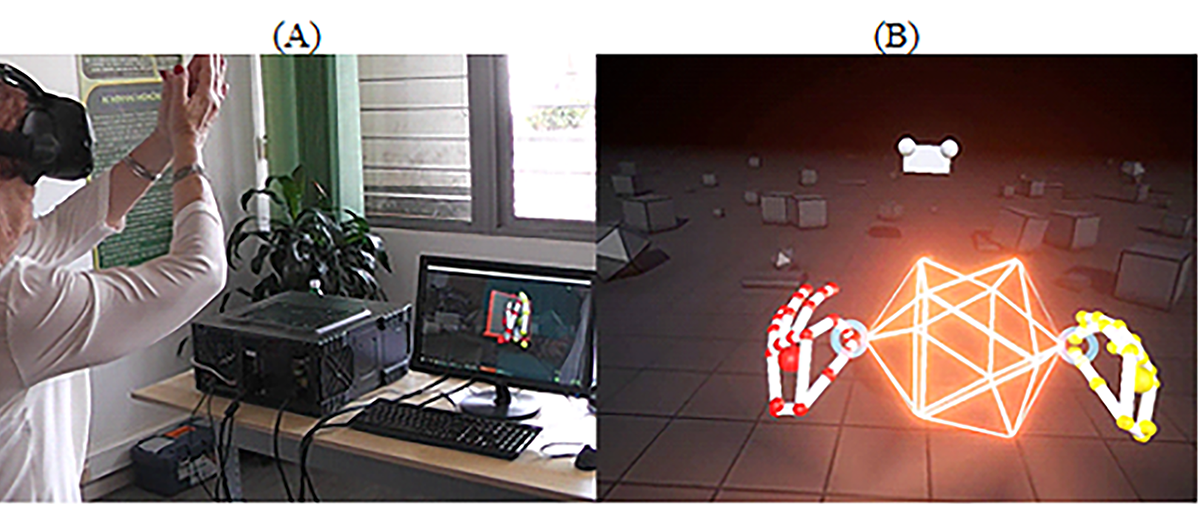
Blocks. (A) Manipulating geometrical figures using hands and head-mounted display with leap motion while standing. (B) Print screen of the game. A set of geometric figures that participants manipulate.

For this task, participants had to interact with virtual cubes using both hands while standing (Figure 4). The game is a free demo included in the LM device [61]. The software allows the creation of different kinds of geometrical figures, such as cubes and octagons. Before the beginning of the task, we prepared the scenario by adding multiple geometrical figures in the virtual environment (Figure 4). The goal of the task was to interact with the geometrical figures by making hand gestures, such as grabbing, throwing, or pushing, among other gestures. Participants could interact with either the right or left hand. Participants were positioned in the middle of the room and could move freely around the room. For security reasons, one researcher was always nearby to aid participants whenever needed. No sounds or music was played during the task, and it did not provide any additional feedback to the participant.

### Playing Musical Instruments

#### LM

**Figure 5:**
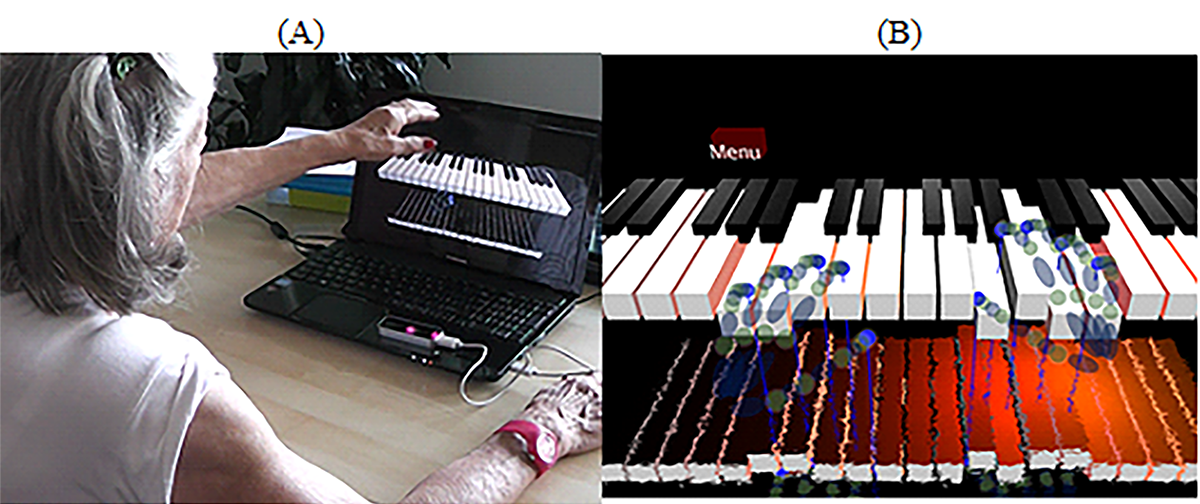
Virtual piano for beginners. (A) Playing the piano using hands and leap motion. (B) Print screen of the game. The virtual piano being played with virtual hands.

The goal of the task was to interact with the piano keyboards using hand movements (Figure 5). The game was a free software included in the LM device [62]. To perform the task, participants had to position their hands above the LM and interact using their fingers. There was no new music playing in the background.

#### HMD With Controllers

**Figure 6:**
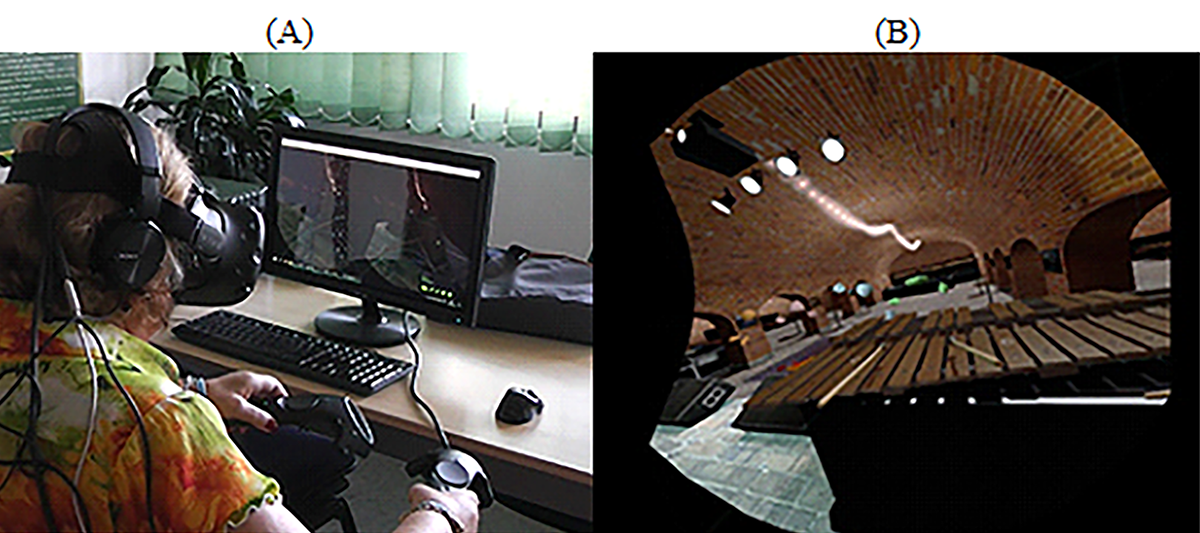
Steam virtual reality home—Playing musical instruments. (A) Playing the xylophone using head-mounted display with controllers. (B) Print screen of the game. Wooden xylophone with wooden sticks and dancing avatars.

We used a virtual environment with a virtual xylophone as, from an interaction perspective, it is very similar to the piano task (Figure 6)*.* We used the free demo of *Jam Session* [60], as it has a variety of instruments, including the xylophone. The goal of the task was to interact with the xylophone while using the HMD headset and handheld controls. To initiate the task, the participant had to (1) grab the controllers with both hands and (2) hit the wooden notes by performing up and down movements with their arms. When interacting with the instrument, dancing avatars would appear in front of the user (Figure 6). Headphones were used by the participants to listen to the sounds while playing the instrument. There was no new music playing in the background.

### Moving Virtual Objects From A to B

#### Mouse

**Figure 7:**
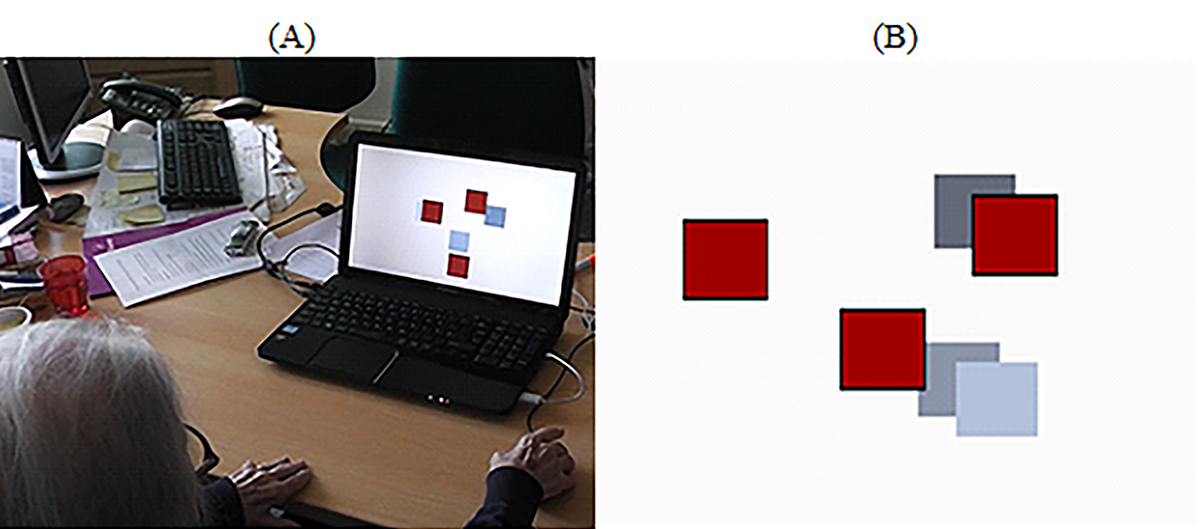
Connecting Squares. (A) Participants are trying to match red squares to gray squares. (B) Print screen of the game. A set of randomly distributed squares.

The goal of the task consisted of pairing—without any order restriction—a set of 3 randomly placed red squares with 3 randomly placed gray squares using a computer mouse device (Figure 7). The game was custom developed using the Unity 3D game engine (Unity Technologies). To complete the task, the participant had (1) to select a red square by pressing the left mouse button (the square becomes green after selection) and (2) select an available gray square by pressing the left mouse button. The right and wheel buttons were deactivated. Audio feedback was provided with “Very Good!” whenever the participant paired all squares.

#### Tablet

**Figure 8:**
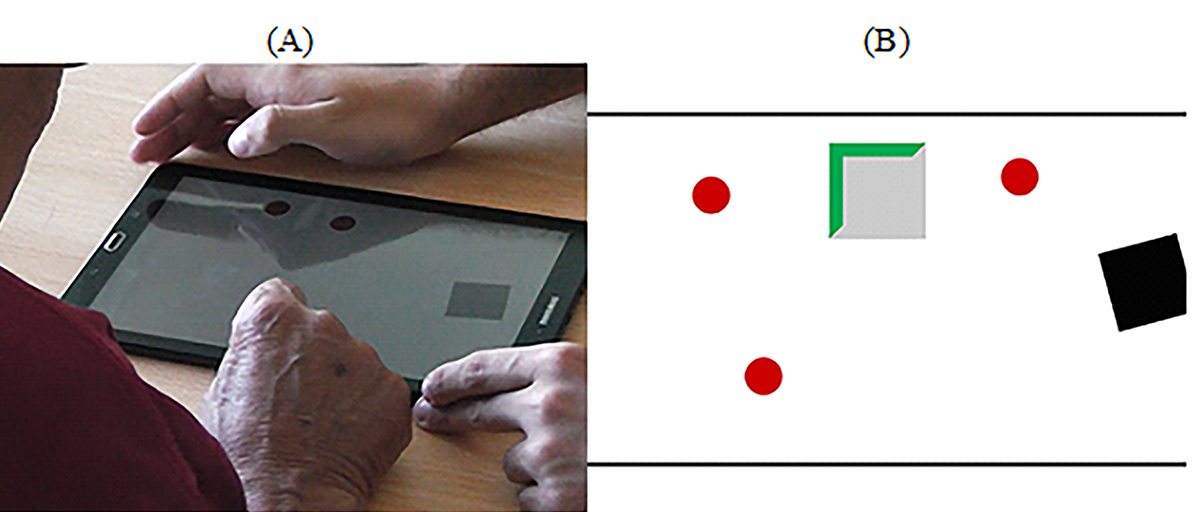
Dragging spheres. (A) Participants are collecting red spheres using a gray container and dragging these with the finger to a black rotating square. (B) Print screen of the game. A set of randomly distributed red spheres, a gray container with the activated timer (in green), and a black rotating target.

In contrast to the previous task using the mouse, the goal of this task was to capture a set of randomly placed red spheres (Figure 8). The game was developed using the Unity 3D game engine (Unity Technologies). To complete the task, participants had to (1) drag a gray container to a red sphere, (2) wait for 4 seconds to attach the sphere to the container, and (3) drag the container with the sphere attached to it to a black rotating target (Figure 8). A countdown sound would provide feedback during the 4-second countdown. After that, the red sphere would become green. In addition, the participant was rewarded with audio feedback—“Very Good”—when all spheres were captured.

#### AR

**Figure 9:**
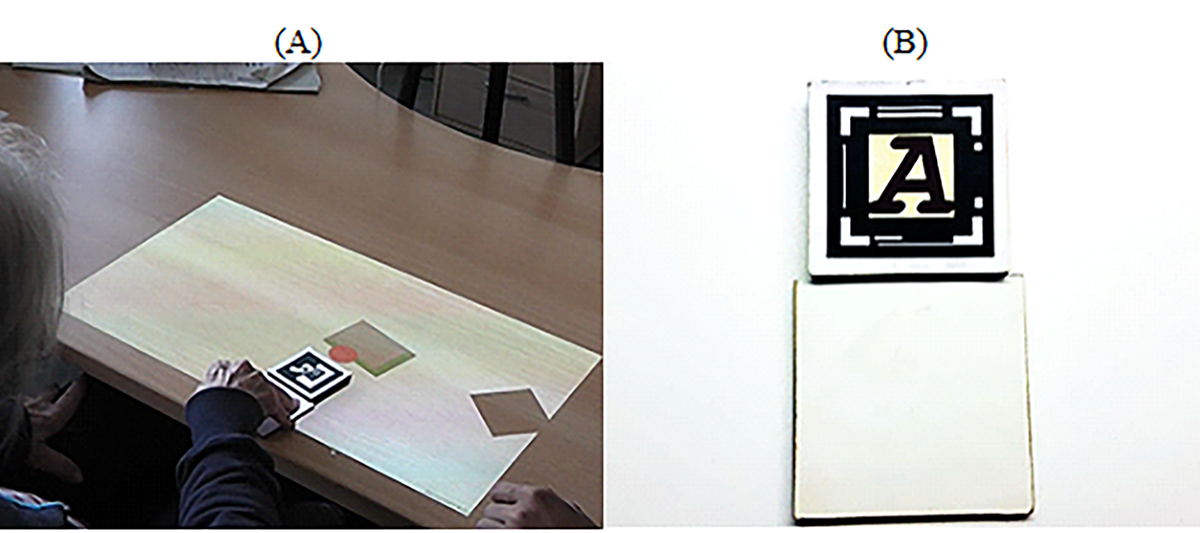
Dragging spheres. (A) Participants are collecting red spheres using a physical object. (B) Physical objects that participants use to interact with the spheres.

For this technology, we used the same task as the one developed for the tablet. However, in this case, participants had to drag a physical object with a gray virtual container attached to it to collect the red spheres and bring them to the black rotating target (Figure 9).

### Observation

#### HMD

**Figure 10:**
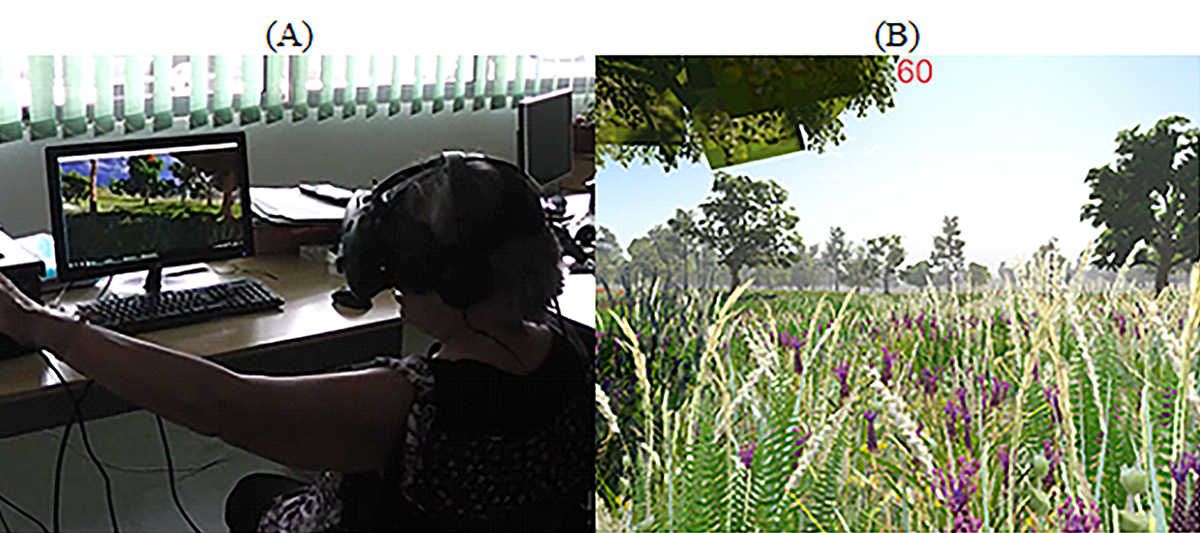
Observing virtual environments: Virtual Forrest. (A) Participants observe a virtual forest using the head-mounted display. (B) Screenshot of the game displaying a virtual forest with trees, grass, and sound.

**Figure 11:**
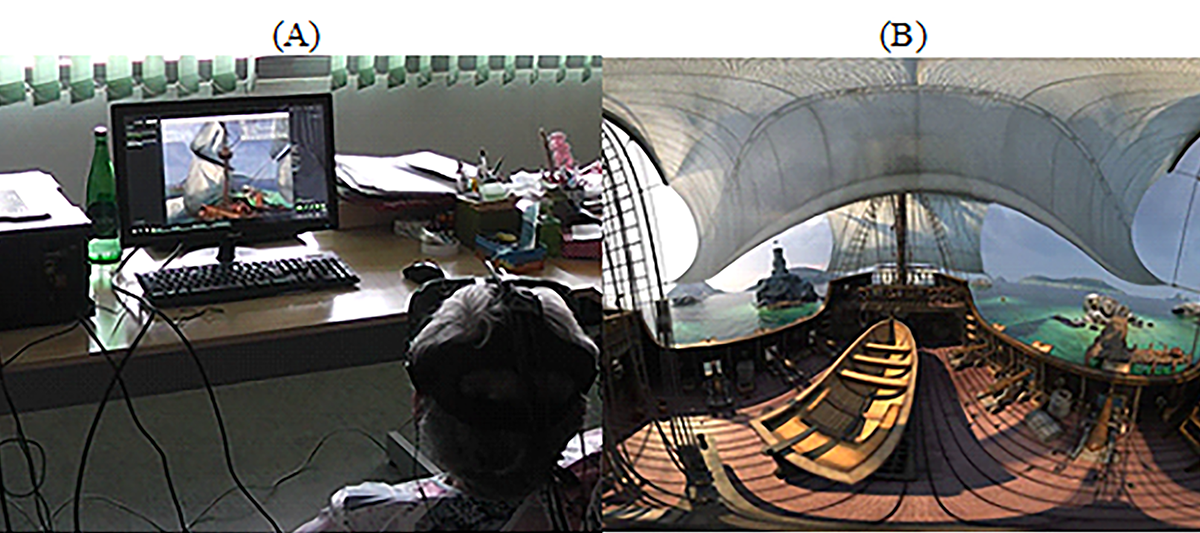
Observing the virtual environments: Ghost Ship. (A) Participants observe an interactive video using the head-mounted display. (B) Screenshot of the Ghost ship sailing in the Caribbean Sea.

In this case, the participants were seated on a chair while wearing the HMD device. The participants were invited to explore virtual worlds by freely moving their heads. We used 2 different tasks to evaluate the participants’ performance:

1. Static scenario—Exploring the forest: In this task, we used a virtual forest that was developed in the Unity 3D game engine (Unity Technologies; Figure 10). It has virtual elements, such as trees, grass, and clouds (among other elements), as well as audible elements, such as birds, insects, and wind (Figure 10). The windy sound effect, combined with the animation of virtual elements, offered dynamism to the scenario by providing the illusion that the virtual elements were moving because of the wind. The goal of this task was to report and describe as many elements as possible.
2. Dynamic scenario—Exploring the ghost ship: This task was a short virtual video of a pirate ship navigating in the natural elements such as rocks, small buildings, and highly detailed pirate ships (Figure 11). The game can be accessed for free on STEAM [63].
  When the video begins, a virtual camera automatically moves on a predefined path while rotating on its axis to show places of interest to the viewer. Generic background music plays in the background, accompanying the participants’ journey. Participants were free to move their head to explore the virtual environment. The goal of the task was to describe and report as many virtual elements as possible to the researchers.

### Procedure

A within-subject experimental design was used to allow all participants to interact with all technologies and tasks. Each week, different technologies and tasks were randomly introduced. Participants were required to complete tasks, such as manipulating virtual objects, moving virtual objects from A to B, observation of virtual scenarios, and playing musical instruments. Participants were seated in a quiet room and accompanied by 2 researchers and a health professional when needed.

During the experiment, patients sat in front of a table in a silent room of the Madeiran delegation of the Portuguese Alzheimer’s Association (except when performing the task requiring a HMD with LM, which in this case required standing up). Two researchers were present in the room; one researcher was responsible for filming and taking notes on the participants’ performance, whereas the other researcher interacted with the participants during the experimental trial. The video recordings allowed us to analyze participants’ behaviors and study their verbal responses throughout the experiment. The camera was placed behind the shoulders of the participants to conceal their faces and protect their identity. In the case of participants in more intermediate stages of dementia, a health professional was also present to guarantee their well-being and aid researchers during the intervention.

During the experiment, 3 protocols were used: (1) before initiating the task, the researchers instructed participants on how to use a specific technology to complete a task; (2) each task had a maximum duration of 15 min, and participants could repeat tasks if desired; and (3) during the task, participants were encouraged to think aloud and could ask for help at any time. All interventions by the researchers were annotated for later analysis.

### Analysis

To address our RQs, we relied on direct observations and behavioral and verbal responses extracted from the video recordings. To analyze the video recordings, we used Adobe Premiere CC 2017.1.2 release version for coding. This video editing tool allowed us to tag, comment, and export annotations in comma-separated values (.CSV) files. The video analysis went through 2 phases. In the first phase, 2 researchers performed independent video analyses by tagging and annotating events in the video files. In the second phase, the information gathered by both researchers was compared and checked for consistency. In case of disagreement, a third researcher was invited to disambiguate.

To analyze participants’ user experience with a given technology or in each task, we counted the number of issues identified. The issues were grouped into (1) assistance provided by researchers, (2) perception issues, (3) comprehension issues, (4) interaction issues, and (5) discomfort that participants felt. These are described in detail below.

1. Assistance provided: We counted the number of times participants required assistance from the researchers. In addition, we considered the assistance provided by therapists if they were present during the experimental session.
2. Comprehension issues: We counted all issues identified in terms of the participants’ general understanding of how to perform the tasks.
3. Perception issues: We considered (1) visual perception issues whenever participants had difficulties in visualizing and correctly identifying game elements during user experience and (2) sensory issues whenever participants had difficulties in hearing and identifying sounds correctly. For (3) tactile issues, we counted the number of times that participants complained of not feeling any physical feedback of the technology during the user experience (ie, lack of vibration and not finding the correct button) and number of times participants were expected to interact with the virtual environment in the same way as in a real-life scenario (ie, expecting to be able to physically touch and feel a virtual object when interacting with it).
4. Interaction issues: We considered issues such as (1) controlling the interface (ie, clicking incorrect buttons to fulfill a task), (2) controlling the software (ie, triggering wrong software functionalities), and (3) the participants physically misusing the interface (ie, grabbing the LM).
5. Discomfort: We counted the number of times participants felt distressed (ie, fatigue, cybersickness, and balance issues).

In addition, we studied emotional responses (positive and negative). That is, we counted the number of positive emotions (ie, laughter) and negative emotions (ie, frustration). We also counted the number of software issues (ie, undesirable features or *bugs*) that occurred during the experiment. For RQ5, we excluded software issues, as these were explicitly related to the actual software and not to the technology per se*.*

Finally, we calculated the number of times the equipment was exposed to external hazards—equipment at risk. For example, we counted the number of times the equipment was at risk of falling to the ground during user experience.

### Data Analysis

Data were analyzed using SPSS, version 24 (IBM Corp). For each dependent variable, the normality of the distribution was assessed using the one-sample Kolmogorov-Smirnov test. As most distributions deviated from normality, nonparametric statistical tests were used for the analysis. Descriptive results are presented as median and IQR. For assessing the impact of experimental conditions, the Friedman test was used. For post hoc pairwise comparisons, the Wilcoxon signed-rank test was used. The significance level was set at α=.05. Bonferroni correction was used to account for multiple comparisons. We also used Bonferroni correction for analyzing which combinations of technologies and tasks minimized feasibility performance (RQ4) and for analyzing which technology was exposed less to external hazards. For nonparametric correlations, we used Spearman rank correlation coefficient.

## Results

### Participants

Participants 1, 2, 3, 7, 11, and 12 completed all 10 experimental conditions. Participants 9 and 10 withdrew from the experiment, and participants 4, 5, 6, and 8 were not able to complete all tasks. Consequently, only 9 datasets were considered for the playground activity with LM; 10 datasets were considered for condition LM (piano activity), tablet, and PC. For AR and observation (exploring the forest), 11 datasets were considered, whereas 7 were considered for condition observation (exploring the ghost ship), playing musical instruments, and manipulating virtual objects with both HTC with controllers and HTC with LM, respectively. In addition, some video recordings were corrupted, which did not allow us to computerize the number of issues; instead, we relied on the written notes taken during the experimental trial.

### Analysis

#### RQ1: Is There a Relationship Between the Patient’s Profile and User Experience?

We studied the relationship between the patients’ profiles when considering each performance domain and each technology separately (Multimedia Appendix 1).

A positive correlation between patients’ MMSE score and perception-related issues when using LM (r_s_=.652; n=10; *P*=.04) was found. We also found a significant and negative correlation between participants’ MMSE scores and the number of assistances provided (r_s_=−.744; n=11; *P*=.01) when using the AR technology. In addition, participants’ years of schooling correlated negatively (r_s_=−.615; n=11; *P*=.04) with perception issues in the AR setup. In terms of the tablet, we found a significant negative correlation in the MMSE scores with both comprehension (r_s_=−.726; n=10; *P*=.02) and interaction issues (r_s_=−.642; n=10; *P*=.045). Finally, for the HMD with LM, we identified a negative correlation between the MMSE scores and the number of assistances provided (r_s_=−.802; n=7; *P*=.03). Multimedia Appendix 2 shows the correlation plots for some of the stronger associations. However, the significance mentioned previously does not endure if adjusted for multiple testing using Bonferroni correction.

Finally, to understand the relationship between the patient profile and performance, we ran a Spearman correlation analysis considering patients’ profile—MMSE, age, schooling, and the total number of issues identified (during user experience)—assistance provided, comprehension issues, interaction issues, perception issues, and discomfort. This analysis did not identify statistically significant correlations between user experience and patient profile (Multimedia Appendix 3).

To identify whether there are any specific tasks or technologies where the cognitive profile may play a role, we repeated the analysis on each task (playing musical instruments, manipulating virtual objects, move objects from A to B, and observation) and each type of technology (LM, HMD, AR, tablet, PC, HMD with controllers, and HMD with LM). Again, we did not find any significant correlations for either task type or technology (Multimedia Appendices 4 and 5).

When considering performance scores by the individual performance domains (ie, assistance provided, discomfort as well as comprehension, interaction, and perception issues), we also found no direct association with the patient’s cognitive profile (Multimedia Appendix 6).

#### RQ2: Is There a Relationship Between User Experience and Direct and Indirect Interaction?

In RQ2, we examined whether there was a difference in participants’ user experience while using direct (LM, AR, tablet, HMD with LM, and HMD) or indirect interaction technologies (HMD with controllers and mouse). In general, participants required less assistance and were able to understand better how to use direct interaction technologies. More concretely, participants required significantly more assistance using indirect interaction devices (median 3.00, IQR 12.00) than using direct interaction devices (median 1.70, IQR 7.00; Z=−2.666; *P*=.01; r=−0.6). Moreover, participants had significantly more comprehension issues with indirect interaction (median 4.00, IQR 5.50) than with the direct interaction (median 2.00, IQR 2.77; Z=−2.601; r=−0.6, *P*=.01). No statistically significant differences were found in interaction issues (median 8.50), perception issues (median 1.50), and discomfort (median 0.40).

#### RQ3: Does Any Technology Elicit More Positive or Negative Emotional Responses?

We evaluated participants’ overall emotional responses while using each technology. For this analysis, we considered the number of positive minus the number of negative emotional reactions identified in the video analysis. There were no statistical differences between the emotional responses and technologies used (χ^2^_6_=7.1; *P*=.31).

#### RQ4: Overall, Which Technology is Better Suited for Each Task?

We analyzed which combinations of technologies and tasks minimized the identified performance and maximized positive emotional reactions. When tasks were grouped by the technology used, participants’ comprehension (χ^2^_6_=23.1; *P*=.001), interaction (χ^2^_6_=19.6; *P*=.003), and discomfort (χ^2^_6_=22.9; *P*=.001) were significantly impacted by technology but not by the number of assistances (median 2.00) and perception issues (median 1.00). A post hoc analysis did not reveal any significant pairwise differences. Table 2 shows the ranking of technology in terms of issues. We ranked the technologies according to their median (IQR).

**Table 2:** Ranking of technologies according to performance domains.

Ranks	Comprehension issues	Interaction issues	Discomfort
First	HMD^a^: 0.00 (0.00)	HMD: 0.00 (0.00)	Mouse: 0.00 (0.00) AR^b^: 0.00 (0.00)
Second	HMD with LM^c^: 0.00 (1.00)	AR: 2.00 (7.00)	N/A^d^
Third	HMD with controllers: 1.00 (5.00)	HMD with LM: 4.00 (6.00)	Tablet: 0.00 (.50)
Fourth	LM: 1.50 (2.50)	HMD with controllers: 6.00 (11.00)	HMD: 0.00 (1.00)
Fifth	AR: 2.00 (3.00)	Mouse: 8.50 (14.50)	HMD with controllers: 0.00 (4.00)
Sixth	Tablet: 4.50 (6.50)	LM: 9.50 (8.25)	LM: 1.00 (3.25)
Seventh	Mouse: 5.00 (5.25)	Tablet: 24.00 (23.00)	HMD with LM: 1.00 (5.00)

^a^HMD: head-mounted display.

^b^AR: augmented reality.

^c^LM: leap motion.

^d^N/A: not applicable. Following a standard competition ranking, there is no device ranking second.

#### Playing Musical Instruments

For this task, we used LM and HMD with controllers, and participants played 2 virtual musical instruments: a piano and a xylophone. Participants showed more perception issues while using the HMD with controllers (median 1.00, IQR 9.00) than when using LM (median 0.00, IQR 0.00; Z=−2.226; r=−0.6, *P*=.03). No other differences between technologies were found.

#### Manipulating Virtual Objects

For this task, participants used the LM, HMD with LM, and HMD with controllers to manipulate a variety of virtual objects. Participants’ performance differed significantly in terms of software issues (χ^2^_2_=6.3; *P*=.04) and equipment at risk (χ^2^_2_=6.5; *P*=.04). We did not find differences in terms of assistance (median 1.00), emotional responses (median −1.00), comprehension (median 0.00), perception (median 0.00), and interaction (median 5.00) issues as well as discomfort (median 1.00). Post hoc analysis revealed no significant pairwise differences among conditions. Table 3 shows the ranking of technologies in the domains in which significant differences were identified. Overall, the combination of HMD with controllers shows a more stable performance in this task. We ranked the technologies according to their median (IQR).

**Table 3:** Ranking of participants’ performance to manipulate objects.

Ranks	Software issues	Equipment at risk
First	HMD^a^ with controllers: 0.00 (0.00)	LM^b^: 0.00 (0.00) HMD with controllers: 0.00 (0.00)
Second	HMD with LM: 1.00 (3.00)	N/A^c^
Third	LM: 1.00 (5.00)	HMD with LM: 1.00 (1.00)

^a^HMD: head-mounted display.

^b^LM: leap motion.

^c^N/A: not applicable. Following a standard competition ranking, there is no device ranking second.

#### Moving Virtual Objects From A to B

For this task, participants used tablet, AR, and mouse devices to move objects from A to B. We found a significant effect of technology in software issues (χ^2^_2_=13.0; *P*=.002) but not in assistance (median 2.00), emotional responses (median 1.00), comprehension issues (median 4.00), interaction issues (median 8.00), perception issues (median 2.00), and discomfort (median 0.00). The technology that raised more software issues was AR (median 2.00, IQR 3.00), followed by tablet (median 0.50, IQR 1.25) and mouse (median 0.00, IQR 0.00). However, no significant pairwise differences were found among them.

#### Observation

**Figure 12:**
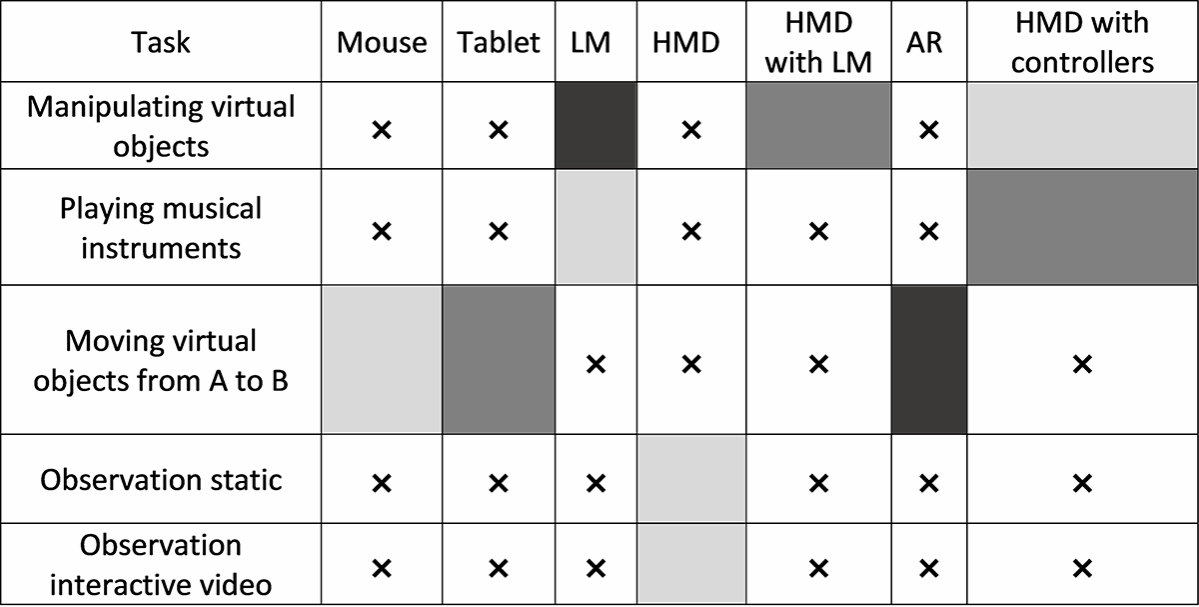
Suitable technologies for each task. Grayscale intensity represents the total number of issues (the lower the intensity, the lower the number of errors), and x represents technologies that have not been used to perform that given task. LM: leap motion; HMD: head-mounted display; AR: augmented reality.

In this task, we studied the impact of 2 modalities: static versus moving content on HMD. Participants explored 2 different environments: a virtual forest and an interactive video. No differences were identified between the 2 modalities. Figure 12 summarizes the findings, reporting the most appropriate technologies by task—manipulating virtual objects, moving virtual objects from A to B, playing musical instruments, and observation.

#### RQ5: Which Technology is the Most Cost-Effective?

One critical factor that may limit the adoption of interactive technologies in this area is their cost. Hence, it is essential to perform a cost-effectiveness analysis to inform therapists and caregivers on the implications of their technological choices in terms of costs and outcomes. In this study, the most expensive technologies were HMD with LM (€578.99 [US $661.76]) and AR (€523.54 [US $598.38]), whereas the cheapest ones were the mouse (€16.99 [US $19.42]), LM (€79.99 [US $91.42]), and tablet (€79.99 [US $91.42]). HMD (€499.00 [US $570.33]), and HMD with controllers (€499.00 [US $570.33]) technology presented a moderate cost. In terms of the (accumulated) identified issues during the study, HMD (46 issues) and HMD with LM (51 issues) had the least issues, whereas tablet presented the most performance issues (433 issues). Technologies such as mouse (209 issues), HMD with controllers (158 issues), LM (166 issues), and AR (193 issues) presented intermediate performance issues. A cost-effectiveness analysis aims to find the right balance that minimizes both cost and number of issues (Multimedia Appendix 7).

We multiplied the number of issues with the purchase price of each technology to calculate the cost efficiency of each technology. The results are presented as the absolute value between the identified issues and costs. As we can see in Multimedia Appendix 7, the most cost-efficient technology is the mouse device, whereas AR is the least cost-efficient technology.

#### RQ6: Which Technology is Less Exposed to External Hazards?

With all the technologies used in this study, we analyzed how they were exposed to risks that could damage the equipment. We found a statistical, but very modest, effect of the type of technology (χ^2^_6_=15.9; *P*=.01). The technology that led to higher risk situations was the HMD with LM (median 1.00, IQR 1.00). However, post hoc pairwise comparisons revealed no significant differences among technologies.

## Discussion

### Comprehension

The technology that ranked best in terms of comprehension was the HMD, whereas technologies that scored worse were mouse and tablet. This is probably because of the simplicity of the interaction with HMDs—participants only need to move their heads to interact with the virtual environments. However, when using the mouse, participants showed great difficulties in understanding how to use it. Most of the difficulties were related to the mapping of the mouse, and sometimes, participants lost sight of the mouse cursor. Participants also had difficulties in interacting with the buttons, being distracted by the mouse wheel many times, as it is the most salient button of the device. Participants tended to rotate and click it instead of using actual mouse buttons. Some participants tried to rotate the mouse wheel forward and backward to move the mouse cursor up and down on the screen. Such behaviors even occurred in participants who had previous professional experience with it. For example, participant 12 had previous experience using the mouse, yet was unsuccessful. As a result, the participant cried, and the experiment had to be stopped. Thus, it becomes crucial to develop intuitive interfaces to avoid overwhelming participants in understanding how to use technology to complete virtual tasks [28].

### Interaction

In terms of interaction, the HMD again ranked the highest. Participants presented issues with the tablet’s interface, mostly because of the multi-touch control. When using multi-touch control, participants would tend to rest their hands on the tablet surface and trigger undesired functions that would prevent them from achieving their goal. Once more, an intuitive software interface is vital to enhance performance in people with dementia [28]. As our tablet was not fixed to the table, it also moved around as participants interacted with it. A better setup would have the tablet fixed to a surface, as in the study by Hackner and Lankes [56]. Despite these issues, the participants were able to perform the task gracefully.

### Discomfort

Concerning discomfort, participants complained the most when using the LM and HMD with LM. For example, participants 1, 2, 10, and 11 reported fatigue while using LM. Indeed, to interact with the LM, participants’ arms need to be moving in the air, leading to muscle fatigue. In the case of the HMD with LM, only participant 5 did not report any discomfort. The remaining participants reported fatigue, cybersickness, and balance difficulties. Although the HMD alone did not trigger major issues, participants 6 and 3 felt nauseated, and participant 12 reported cybersickness after the virtual video task. Participant 6 complained about the heat generated by the headset. In general, cybersickness and fatigue are some of the negative aspects identified in the scientific literature in terms of the use of technology, whereas balance-related issues are associated with the negative consequences of dementia [15,17,28,55].

### Effect of Patient Profile

We found that the participants’ profile influences the usage of technology. A negative and significant correlation between MMSE scores and the number of assistances provided with AR and HMD with LM were found. In the case of AR technology, we found a significant effect on the level of schooling and the number of perception issues that arose in the experiment. We also saw that a low level of schooling and lack of experience with novel technology could lead to confusion (or even anxiety) [54]. For example, participant 3 was confused when instructed to move the red spheres that were projected on the table; as a result, the participant questioned: “*How can I catch the spheres if they are fixed on a table?”* Concerning the usage of the tablet, we found a significant correlation between MMSE scores and both comprehension and interaction issues. This is likely because of the multi-touch feature. Some participants failed to understand that by placing the whole hand on the screen of the tablet, multi-touch is triggered. Other issues that were identified included (1) activating the menu buttons of the tablet involuntarily, (2) dragging the tablet involuntarily while interacting with the virtual objects, (3) forgetting to wait for the selection time, and (4) forgetting the task rules. Finally, we found a positive and significant correlation between the MMSE scores and the number of perception issues when using LM. We observed that participants with high MMSE scores were able to interact with technology easily and for longer, which allowed researchers to identify perception issues during user experience, in contrast to participants with lower MMSE scores who struggled to begin a given task. Similar results were found in the study by Alvseike and Brønnick [44], which found that individuals with higher cognitive deficits had more difficulties in using smart house technology than individuals with lower cognitive deficits. Performance may also depend on other variables, such as motivation and experience [43].

### Direct Versus Indirect Interaction

Participants required more assistance statistically and had more difficulties in understanding how to use indirect interaction devices. Indirect interaction devices require more cognitive resources [41] and, in a population with cognitive deficits, may hinder performance during the completion of tasks. Conversely, direct interaction devices require less cognitive resources, and, consistent with our observations, participants had fewer complications in using such technologies as they are more intuitive and straightforward to interact with virtual content. Some participants, such as participants 1 and 11, were able to use both direct and indirect interaction technologies with minor problems. However, it is important to take into consideration that these participants had higher MMSE scores, and that participant 11 had experience in using mouse technology.

### Emotional Responses

Participants, in general, did not show many emotional responses when using the studied technologies. However, some interesting reactions were observed. For example, participant 1 was very happy when she was able to grab a cube while using the HMD with LM and said, “*Oh good...what a funny thing...it is so beautiful.*” The same participant showed pride while playing the xylophone with the HMD with controllers and said that it was a shame that the people in the room could not hear her playing as it was a beautiful song. Participant 11 enjoyed exploring virtual environments with the HMD. She repeatedly said “*very beautiful*” in both the *Exploring the Forest* and *Exploring the Ghost Ship* tasks.

### Playing Musical Instruments

Here, participants used the LM and HMD with controllers to play virtual instruments and showed more perception issues while using the HMD with controllers than LM. Most of the issues identified were visual, auditory, and tactile related. For example, participant 12 complained that she did not hear the xylophones (yet, she confirmed during the experience that she heard the sounds). The same participant also reported that she was not able to see anything several times. In addition, participant 3 complained that she was not able to see or reach the musical instruments (despite being within the participant’s arm range).

### Manipulating Virtual Objects

In this task, the participants used the LM, HMD with LM, and HMD with controllers to manipulate virtual objects. We found differences in terms of software issues and equipment at risk. In general, the best technology is HMD with controllers. Although there were no statistically significant perception issues, participant 12 raised most visual-related problems, as she had difficulties in identifying the virtual objects in the virtual environments, including the digital representation of her hand. Participant 3 complained because she was expecting to “physically” grab the virtual objects. In terms of software issues, the HMD with controllers scored first place as it did present minor issues.

In contrast, LM technology scored the worst (last place). As participants tried to grab virtual objects, sometimes the objects stayed attached involuntarily to their hands, and they struggled to let go of the objects. Similar behaviors were recorded while participants performed the task while using the HMD with LM. Participants were able to *grab* virtual objects but had more difficulties dropping them. Finally, in terms of equipment at-risk situations, the HMD with LM triggered more dangerous situations for the equipment. For example, when participants were performing abrupt movements with the head, the HMD was sometimes at risk of falling.

### Move Objects From A to B

We only found a statistical difference in terms of software issues*,* with AR and tablet being the ones that scored the worst. AR technology had some camera tracking issues because of environmental issues, such as shadows and reflections. In contrast, most of the issues related to the tablet were because of software bugs. Despite these minor issues, all technologies performed at an acceptable level.

### Observation

In this task, we did not find any statistical differences. The only issues identified were related to cybersickness in both observation tasks [55].

### Design Recommendations

In this study, we observed that technology had different outcomes in terms of acceptance and performance on people with dementia. Although technologies have been accepted by the majority, some participants had difficulties in managing them to fulfill the tasks. Such differences in the results are mainly because of patient profiles, which, in turn, influence technology configuration (direct interaction versus indirect interaction).

Comprehensibly, most of the technologies used were not aligned with the REAFF framework, as these were not explicitly designed to take into consideration the needs of people with dementia [45,46]. Most of the technologies used did not follow, for example, the augmenting or failure free principles, as participants did not complete the tasks independently. It is also important to consider how to align such technologies with the remaining principles of the REAFF framework for the needs of people with dementia (responding) and how technology can improve their everyday life (enabling).

In addition, it would be interesting to re-evaluate such technologies using similar tasks as presented in this study, but in a clinical context using the VR-Check framework [47]. Thus, more detailed knowledge could be gained regarding the adequacy and therapeutic outcome when using technology and virtual reality with people with dementia.

Although the technologies used are not perfectly aligned with the REAFF framework principles, they are accessible and can be used in their favor if they are set up correctly. By studying the use of the different technologies and tasks by people with dementia, we can provide a set of recommendations for the selection and implementation of different technological solutions when working with this population. Table 4 addresses the main problems encountered and provides recommendations to overcome them. These recommendations can help engineers in the design of technologies for people with dementia and draw attention to health professionals and informal caregivers regarding potential issues that can emerge while using such technologies with this population.

**Table 4:** Identification of problems and proposed recommendations or using technology to perform virtual tasks by people with dementia.

Technology, Identified problems		Solution
**LM^a^**		
	Grabbing/moving technology needlessly	Design a setup where the LM is fixed and not graspable (ie, a 3D printed container or embedded onto the tabletop surface).
	Confusing virtual objects (spheres) with the joints of the virtual hand	Use identifiable virtual objects and representations of the hand with higher realism.
**Tablet**		
	Moving the whole tablet involuntarily	Secure the tablet on a table or fixed structure such that patients do not need to hold it and can interact with its touch screen.
	Triggering undesired touch inputs	Deactivate multi-touch and disable system buttons.
**AR^b^**		
	Interaction with physical elements	Ergonomic design with affordances consistent with the task at hand can enhance performance.
	Tracking problems	The most common tracking problems are related to (1) shadows, (2) markers out of the camera’s field-of-view, or (3) projection of virtual elements on markers. Solutions include using a room without direct sunlight and controlled light conditions; using lower contrast virtual elements that diminish interference of projecting on markers; and using a setup with clearly defined interaction boundaries.
**Mouse**		
	Buttons not salient	Select a computer mouse that visually clearly identifies where those buttons are. A large colored sticker or paint on a button can also be used to improve its saliency.
	Too many buttons	Most modern computer mice consist of 3 buttons and a scroll wheel. Choose a one-button mouse (ie, Apple mouse). Disabling or mapping all mouse buttons to the same functionality will minimize the impact of choosing the wrong button.
	Mouse cursor (and other elements) too small	Increase the size of the mouse cursor and other virtual elements to enhance performance.
**HMD^c^** **with controllers**		
	Too many buttons in handheld controls	Users only see a virtual representation of the controls in the HMD. Minimum button input should be considered while the remaining buttons are disabled or mapped to the same function.
	Hitting controls against each other	Use only one control to interact with the virtual content when possible. Alternatively, replace the controllers with an LM.
**HMD with LM**		
	Lack of haptic feedback	Complement with alternative channels to convey haptic feedback (ie, auditory or visual).
	Cybersickness and balance issues	People with disabilities need to be assessed for balance, and seating setups should be considered. Safety harnesses or other safety measures should be considered when standing.
**HMD**		
	Discomfort because of the device’s heat	Use in a properly ventilated room. In case of discomfort, divide the session into multiple shorter intervals.
	Cybersickness	Virtual environments should be designed to minimize optic flow, and incongruency between physical and virtual motion should be minimized. It can be achieved by reducing forward motion and rotations as well as using simpler environments with fewer visual elements.

^a^LM: leap motion.

^b^AR: augmented reality.

^c^HMD: head-mounted display.

### Conclusions

This study involved 12 participants with dementia who performed 5 different tasks using 5 interactive technologies that were available at the time of this study. As participants used the technologies to perform virtual reality tasks, we identified potential issues, such as assistance provided, comprehension issues, perception issues, interaction issues, and discomfort. We also studied how the patient’s profile would affect performance in those different tasks and technologies. Finally, we provided a set of recommendations for the selection, use, and design of virtual tasks for these technologies. Our main findings show significant effects of technology on performance regarding comprehension, interaction, and discomfort.

Overall, the participants were able to complete all tasks using all technologies. However, a clear outcome of the study is that there is no absolute best technology for people with dementia, but this is both task- and patient-profile–dependent. In general, the use of technologies that require direct interaction is advisable, given that cognitive performance gradually declines in people with dementia, as it relies on fewer cognitive resources than indirect interaction devices. We observed that cognitive skills, as assessed by the MMSE test, influenced participants’ perception, comprehension*,* and interaction and required more assistance*.* In addition, schooling is also a factor to be considered; the lack of experience and exposure to such technologies can lead to confusion and anxiety, which interferes with user experience.

A cost-effectiveness analysis comparing price and issues identified in all technologies suggests that the best tradeoff between performance and cost is achieved with the mouse, the most effective technology is HMD, and the most expensive one is AR. Through these insights, this study provides newer insights for health professionals, informal caregivers, and engineers regarding the use and design of novel technologies for people with dementia to (1) maximize their success in using such technologies to fulfill virtual tasks and (2) safeguard their psychological well-being. The findings of this study and the proposed guidelines are being implemented on a set of SGs for cognitive stimulation, which explores the potential benefits of music and reminiscence-related approaches in people with dementia.

To conclude, the participants in this study were able to handle the technologies to complete virtual tasks. Interestingly, the overall success in using technologies by people with dementia depends on different variables, such as patient profile, type of task, and interaction modality. Our study provides a quantitative analysis that contributes toward a better understanding of the complex relationships among these factors. Finally, by translating our findings into a set of guidelines, we hope to facilitate technological interventions and to enhance the user experience of people with dementia when performing virtual tasks with *out-of-the-shelf* technologies.

### Limitations

This study has some limitations. We had a small number of participants, and not all participants interacted with all technologies. Consequently, if we applied Bonferroni correction for multiple comparisons, statistical significances during post hoc analysis do not remain. Hence, a larger sample size would have provided higher statistical power for the analysis. In addition, having a control group of healthy age- and sex-matched participants would have been informative to discriminate age- and dementia-related issues, such as perception problems. Future studies should consider adding a control group to draw additional conclusions regarding the usage of *out-of-the-shelf* technologies to perform virtual reality tasks. Nevertheless, adding a control group presented some challenges.

First, we interacted with a population that cannot adequately express themselves in the same way as healthy elderly people. Therefore, we had to use very time-consuming methodologies, such as independent annotation of hours of video recordings, categorization, and extraction of data, so that a quantitative analysis could be performed. Consequently, we would have to use the same methodology as the control group (that would not require it), making it not feasible for us, given the time needed and available human resources. Second, even if we did so, our experience tells us that the 2 groups would not be directly comparable even if performing the same activities because people with dementia required constant stimulation and assistance by researchers and health professionals to understand and perform the tasks. Third, the level of autonomy of people with dementia in performing the activities is not comparable with that of a healthy old adult.

In addition, when performing the cost-effectiveness analysis, we considered different approaches, such as the normalization of costs and issues. However, we realized that the resulting values obscured the actual relation to either cost or actual issues, making it very difficult to interpret. Moreover, we considered performing an *issues per euro* analysis; however, such an approach was also problematic because the metric favored expensive equipment. That is, the more expensive the equipment, the less the issues and cost ratio. Similarly, very cheap equipment, such as the mouse, always presents a very high (comparatively) issue/cost ratio. Therefore, in the cost-effectiveness analysis, we gave the same weight to issues and cost because (1) it is fairer to compare and (2) easier to interpret.

Moreover, the use of assessment tools should be considered for additional qualitative data analysis, such as the Individually Prioritized Problem Assessment and the Psychosocial Impact of Assistive Devices Scale, to evaluate how technology impacts the daily life of people with dementia. Finally, some video recordings were corrupted, and, although we also used written notes, some level of detail may have been lost.

## Appendix

Multimedia Appendix 1 Correlation between participants profile performance and technology.

Multimedia Appendix 2 Scatter plots.

Multimedia Appendix 3 Correlations between patient profile and overall technology.

Multimedia Appendix 4 Correlation between patient profile and task.

Multimedia Appendix 5 Correlation between participants profile and performance with different technologies.

Multimedia Appendix 6 Correlation between participants profile and individual performance domain.

Multimedia Appendix 7 Relationship between performance issues that emerged while performing tasks with different technologies and purchase costs of technology. The price (euros) was that of the equipment when it was purchased.

## Abbreviations

AR: augmented reality
HMD: head-mounted display
LM: leap motion
MMSE: Mini-Mental State Examination
REAFF: responding, enabling, augmenting, failure free
RQs: research questions
SG: serious game
VR: virtual reality
