# Supplementary material for: User Experience of Interactive Technologies for People With Dementia: Comparative Observational Study
Source: JMIR Serious Games. 2020 Aug 5;8(3):e17565. doi: 10.2196/17565 (PMC7439148; doi:10.2196/17565)
Supplement: Multimedia Appendix 1 [file games_v8i3e17565_app1.docx]

|  | | | | | |
| --- | --- | --- | --- | --- | --- |
|  | Leap Motion | | | | |
| **Participants**  **Profile** | Assistance | Comprehension | Interaction | Perception | Discomfort |
| MMSE | r_s_ = -.445,  n = 10,  p = .197 | r_s_ = -.549,  n = 10,  p = .101 | r_s_ = -.152,  n = 10,  p = .675 | r_s_ **= .652*,**  **n = 10,**  **p = .041** | r_s_ = -.057,  n = 10,  p = .876 |
| Age | r_s_ = -.049,  n = 10,  p = .894 | r_s_ = .362,  n = 10,  p = .305 | r_s_ = -.122,  n = 10,  p = .738 | r_s_ = -.090,  n = 10,  p = .805 | r_s_ = .374,  n = 10,  p = .287 |
| Schooling | r_s_ = -.058,  n = 9,  p = .883 | r_s_ = -.297,  n = 9,  p = .437 | r_s_= -.282,  n = 9,  p = .462 | r_s_ = -.394,  n = 9,  p = .294 | r_s_ = .482,  n = 9,  p = .189 |
|  | | | | | |
|  | HMD w/ Controllers | | | | |
| **Participants**  **Profile** | Assistance | Comprehension | Interaction | Perception | Discomfort |
| MMSE | r_s_ = -.617,  n = 7,  p = .140 | r_s_ = -.296,  n = 7,  p = .518 | r_s_ = -.036,  n = 7,  p = .939 | r_s_ = -.296,  n = 7,  p = .518 | r_s_ = -.438,  n = 7,  p = .325 |
| Age | r_s_ = -.168,  n = 7,  p = .718 | r_s_ = .259,  n = 7,  p = .574 | r_s_ = .179,  n = 7,  p = .702 | r_s_ = .259,  n = 7,  p = .574 | r_s_ = -.259,  n = 7,  p = .575 |
| Schooling | r_s_ = .021,  n = 7,  p = .965 | r_s_= -.225,  n = 7,  p = .628 | r_s_ = -.453,  n = 7,  p = .307 | r_s_ = -.225,  n = 7,  p = .628 | r_s_ = -.055,  n = 7,  p = .907 |
|  | | | | | |
|  | HMD | | | | |
| **Participants**  **Profile** | Assistance | Comprehension | Interaction | Perception | Discomfort |
| MMSE | r_s_ = -.035,  n = 11,  p = .919 | r_s_ = -.419,  n = 11,  p = .200 | r_s_ = -.200,  n = 11,  p = .555 | r_s_ = -.425,  n = 11,  p = .193 | r_s_ = -.304,  n = 11,  p = .363 |
| Age | r_s_ = -.244,  n = 11,  p = .470 | r_s_ = -.405,  n = 11,  p = .217 | r_s_= .000,  n = 11,  p = 1.000 | r_s_ = -.189,  n = 11,  p = .578 | r_s_ = -.249,  n = 11,  p = .461 |
| Schooling | r_s_ = .314  n = 10,  p = .376 | r_s_ = .072,  n = 10,  p = .844 | r_s_ = -.482,  n = 10,  p = .158 | r_s_= .401,  n = 10,  p = .251 | r_s_ = -.186,  n = 10,  p = .607 |
|  | | | | | |
|  | Augmented Reality | | | | |
| **Participants**  **Profile** | Assistance | Comprehension | Interaction | Perception | Discomfort |
| MMSE | r_s_ **= -.744*,**  **n = 11,**  **p = .009** | r_s_ = -.324,  n = 11,  p = .331 | r_s_ = .196,  n = 11,  p = .563 | r_s_ = .406,  n = 11,  p = .215 | r_s_ = -.351,  n = 11,  p = .290 |
| Age | r_s_ = -.066,  n = 11,  p = .848 | r_s_ = -.070,  n = 11,  p = .837 | r_s_ = .078,  n = 11,  p = .819 | r_s_ = .221,  n = 11,  p = .513 | r_s_ = -.500,  n = 11,  p = .117 |
| Schooling | r_s_ = .035,  n = 11,  p = .919 | r_s_ = -.237,  n = 11,  p = .483 | r_s_ = -.423,  n = 11,  p = .195 | r_s_ **= -.615*,**  **n = 11,**  **p = .044** | r_s_ = .474,  n = 11,  p = .140 |
|  | | | | | |
|  | Tablet | | | | |
| **Participants**  **Profile** | Assistance | Comprehension | Interaction | Perception | Discomfort |
| MMSE | r_s_ = -.620,  n = 10,  p = .056 | r_s_ **= -.726,***  **n = 10,**  **p = .017** | r_s_ **= -.642,***  **n = 10,**  **p = .045** | r_s_ = -.182,  n = 10,  p = .615 | r_s_ = -.261,  n = 10,  p = .466 |
| Age | r_s_ = -.141,  n = 10,  p = .697 | r_s_ = -.037,  n = 10,  p = .919 | r_s_ = -.220,  n = 10,  p = .541 | r_s_ = .151,  n = 10,  p = .678 | r_s_ = .000,  n = 10,  p = 1.000 |
| Schooling | r_s_= .066,  n = 9,  p = .867 | r_s_ = .198,  n = 9,  p = .610 | r_s_ = .033,  n = 9,  p = .934 | r_s_ = .068,  n = 9,  p = .863 | r_s_ = -.530,  n = 9,  p = .142 |
|  | | | | | |
|  | PC | | | | |
| **Participants**  **Profile** | Assistance | Comprehension | Interaction | Perception | Discomfort |
| MMSE | r_s_ = -.468,  n = 10,  p = .173 | r_s_ = .108,  n = 10,  p = .766 | r_s_ = -.367,  n = 10,  p = .297 | r_s_ = .416,  n = 10,  p = .232 | r_s_ = -  n = 10,  p = - |
| Age | r_s_ = .129,  n = 10,  p = .723 | r_s_ = -.092,  n = 10,  p = .800 | r_s_= .317,  n = 10,  p = .372 | r_s_ = -.343,  n = 10,  p = .332 | r_s_ = -  n = 10,  p = - |
| Schooling | r_s_ = -.211,  n = 10,  p = .559 | r_s_ = -.575,  n = 10,  p = .082 | r_s_ = -.269,  n = 10,  p = .453 | r_s_ = .217,  n = 10,  p = .547 | r_s_ = -  n = 10,  p = - |
|  | | | | | |
|  | HMD w/ LM | | | | |
| **Participants**  **Profile** | Assistance | Comprehension | Interaction | Perception | Discomfort |
| MMSE | r_s_ **= -.802*,**  **n = 7,**  **p = .030** | r_s_= .045,  n = 7,  p = .924 | r_s_ = -.464,  n = 7,  p = .294 | r_s_ = -.519,  n = 7,  p = .233 | r_s_ = -.449,  n = 7,  p = .312 |
| Age | r_s_= -.535,  n = 7,  p = .216 | r_s_ = .134,  n = 7,  p = .775 | r_s_ = -.107,  n = 7,  p = .819 | r_s_ = .037,  n = 7,  p = .937 | r_s_ = -.056,  n = 7,  p = .905 |
| Schooling | r_s_ = .516,  n = 7,  p = .236 | r_s_ = -.172,  n = 7,  p = .712 | r_s_= -.079,  n = 7,  p = .867 | r_s_= .123,  n = 7,  p = .793 | r_s_ = -.206,  n = 7,  p = .657 |
|  | | | | | |
