# Supplementary figures and images for: User Experience of Interactive Technologies for People With Dementia: Comparative Observational Study
Source: JMIR Serious Games. 2020 Aug 5;8(3):e17565. doi: 10.2196/17565 (PMC7439148; doi:10.2196/17565)

| 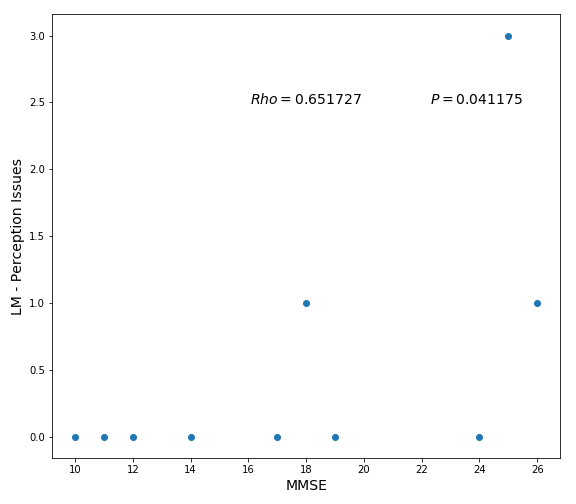 | 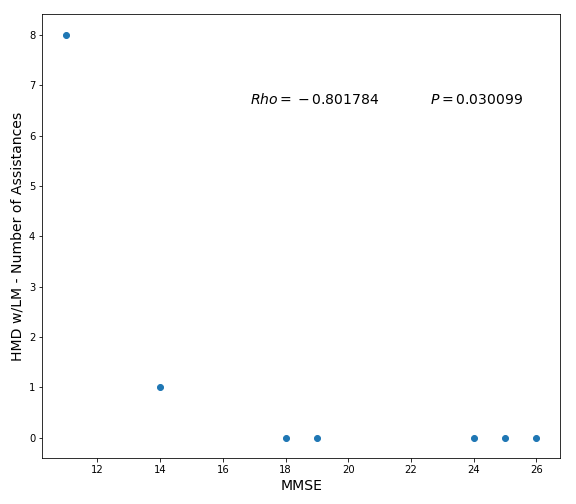 |
| --- | --- |
| 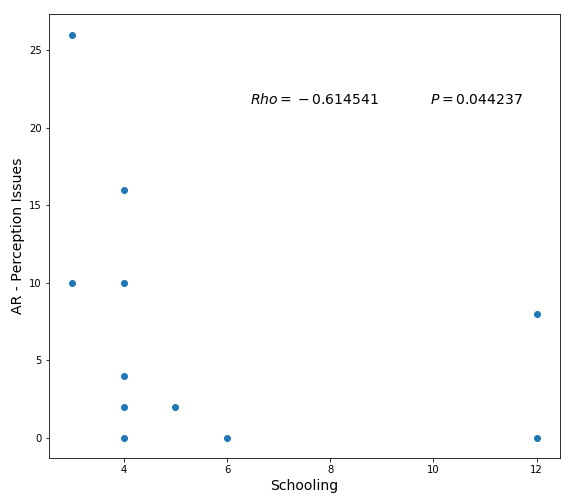 | 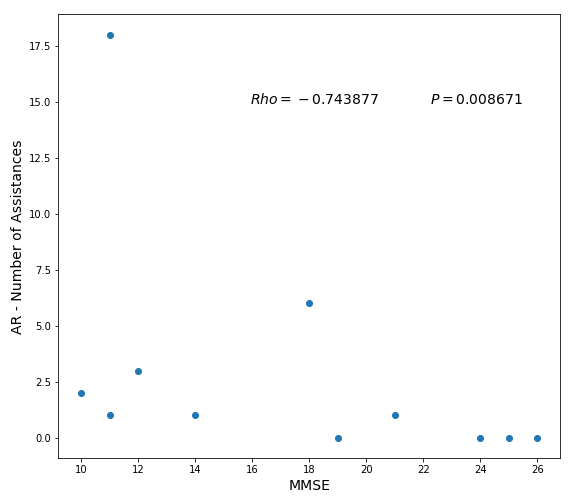 |
| 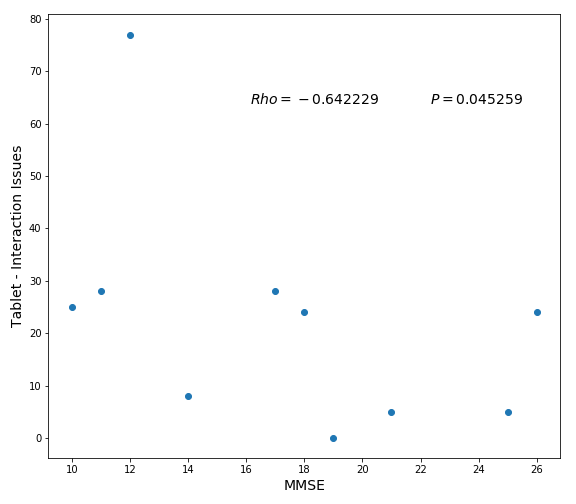 | 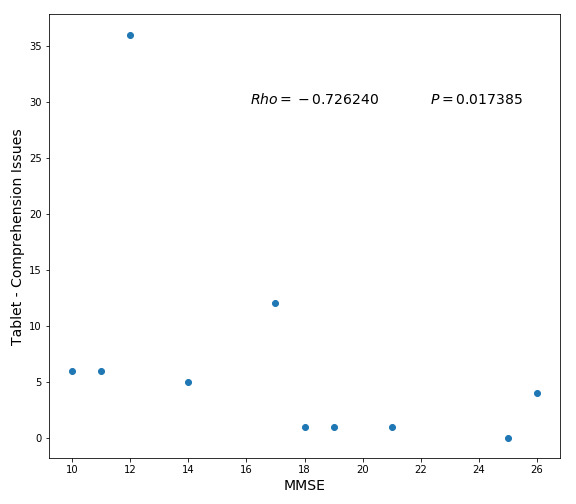 |
|  | |

Supplement: Multimedia Appendix 2 [file games_v8i3e17565_app2.docx]

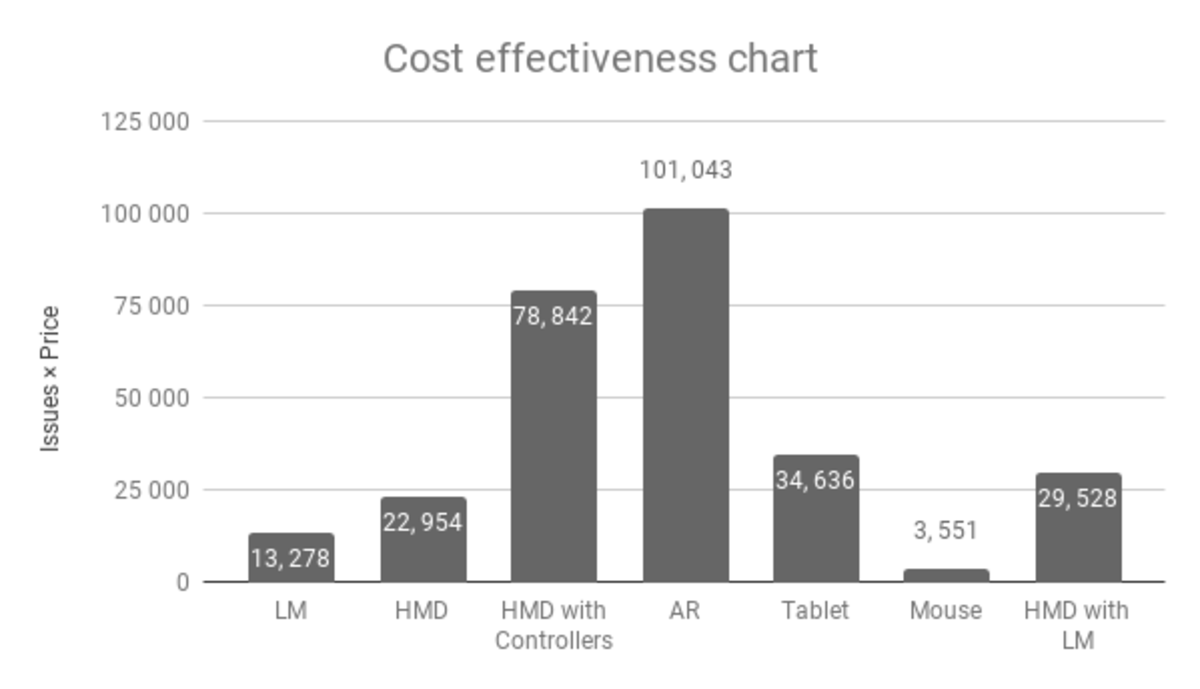

Supplement: Multimedia Appendix 7 [file games_v8i3e17565_app7.png]
