# Supplementary material for: User Experience of Interactive Technologies for People With Dementia: Comparative Observational Study
Source: JMIR Serious Games. 2020 Aug 5;8(3):e17565. doi: 10.2196/17565 (PMC7439148; doi:10.2196/17565)
Supplement: Multimedia Appendix 3 [file games_v8i3e17565_app3.docx]

|  | |
| --- | --- |
| Patient Profile | Technology and User Experience |
| MMSE | r_s_ = -.235, n = 12, p = .463 |
| Age | r_s_ = .084, n = 12, p = .795 |
| Schooling | r_s_ = -.302, n = 11, p = .367 |
