# Supplementary material for: User Experience of Interactive Technologies for People With Dementia: Comparative Observational Study
Source: JMIR Serious Games. 2020 Aug 5;8(3):e17565. doi: 10.2196/17565 (PMC7439148; doi:10.2196/17565)
Supplement: Multimedia Appendix 4 [file games_v8i3e17565_app4.docx]

|  |  | | | |
| --- | --- | --- | --- | --- |
|  | Tasks | | | |
| Participant Profile | Playing Musical Instruments | Manipulating Virtual Objects | Move Objects from A to B | Observation |
| MMSE | r_s_ = -.105, n = 10,  p = .774 | r_s_= -.0.17, n = 9,  p = .966 | r_s_= -.329, n = 12, p = .296 | r_s_ = -.397, n = 11,  p = .226 |
| Age | r_s_ = .197, n = 10,  p = .586 | r_s_= -.277, n = 9,  p = .470 | r_s_ = .070, n = 12, p = .829 | r_s_ = -.258, n = 11,  p = .444 |
| Schooling | r_s_ = -.233, n = 9,  p = .546 | r_s_= -.292, n = 9,  p = .445 | r_s_ = -.264, n = 11, p = .434 | r_s_ = .077, n = 10,  p = .833 |
