# Supplementary material for: User Experience of Interactive Technologies for People With Dementia: Comparative Observational Study
Source: JMIR Serious Games. 2020 Aug 5;8(3):e17565. doi: 10.2196/17565 (PMC7439148; doi:10.2196/17565)
Supplement: Multimedia Appendix 5 [file games_v8i3e17565_app5.docx]

|  | | | | | | | |
| --- | --- | --- | --- | --- | --- | --- | --- |
|  | Technology | | | | | | |
| Participant Profile | LM | HMD | AR | Tablet | PC | HMD w/ Controllers | HMD w/ LM |
| MMSE | r_s_ = -.334, n = 10,  p = .345 | r_s_ = -.397,  n = 11,  p = .226 | r_s_= .025,  n = 11,  p = .941 | r_s_ = -.394,  n = 10,  p = .260 | r_s_ = -.284,  n = 10,  p = .426 | r_s_ = -.250,  n = 7,  p = .589 | r_s_ = -.559, n = 7,  p = .192 |
| Age | r_s_ = -.006, n = 10  p = .987 | r_s_ = -.258,  n = 11,  p = .444 | r_s_ = .160,  n = 11,  p = .638 | r_s_ = -.164,  n = 10,  p = .651 | r_s_ = .177,  n = 10,  p = .625 | r_s_ = .036,  n = 7,  p = .939 | r_s_ = -.126, n = 7,  p = .788 |
| Schooling | r_s_ = -.084,  n = 9,  p = .830 | r_s_ = .077 n = 10,  p = .833 | r_s_ = -.439, n = 11,  p = .177 | r_s_ = -.065, n = 9,  p = .869 | r_s_ = -.288,  n = 10,  p = .419 | r_s_ = -.315,  n = 7,  p = .491 | r_s_ = -.0.80,  n = 7,  p = .865 |
