# Supplementary material for: User Experience of Interactive Technologies for People With Dementia: Comparative Observational Study
Source: JMIR Serious Games. 2020 Aug 5;8(3):e17565. doi: 10.2196/17565 (PMC7439148; doi:10.2196/17565)
Supplement: Multimedia Appendix 6 [file games_v8i3e17565_app6.docx]

|  | | | | | |
| --- | --- | --- | --- | --- | --- |
|  | Individual Performance domain | | | | |
| **Participants**  **Profile** | Assistance | Comprehension | Interaction | Perception | Discomfort |
| MMSE | r_s_ = -.452,  n = 12,  p = .140 | r_s_ = -.306,  n = 12,  p = .334 | r_s_ = -.217,  n = 12,  p = .498 | r_s_ = .067,  n = 12,  p = .837 | r_s_= -.092,  n = 12,  p = .776 |
| Age | r_s_ = .028,  n = 12,  p = .931 | r_s_ = .004,  n = 12,  p = .991 | r_s_ = .175,  n = 12,  p = .587 | r_s_ = .179,  n = 12,  p = .579 | r_s_ = .046,  n = 12,  p = .887 |
| Schooling | r_s_ = -.125,  n = 11,  p = .715 | r_s_ = -.355,  n = 11,  p = .283 | r_s_ = -.388,  n = 11,  p = .238 | r_s_= -.264,  n = 11,  p = .433 | r_s_= -.027,  n = 11,  p = .938 |
